# Supplementary material for: A multi-phenotype analysis reveals 19 susceptibility loci for basal cell carcinoma and 15 for squamous cell carcinoma
Source: Nat Commun. 2022 Dec 10;13:7650. doi: 10.1038/s41467-022-35345-8 (PMC9741635; doi:10.1038/s41467-022-35345-8)
Supplement: Supplementary file 1 — Supplementary Information [file 41467_2022_35345_MOESM1_ESM.pdf]

Supplementary information

A multi-phenotype analysis reveals 19 susceptibility loci for basal cell carcinoma and 15 for squamous cell carcinoma.

Mathias Seviiri*[1,2,3], Matthew H. Law[1,2], Jue-Sheng Ong[1], Puya Gharahkhani[1], Pierre Fontanillas[4], The 23andMe Research Team[#4], Catherine M. Olsen[5,6], David C. Whiteman[6] and Stuart MacGregor [1,2]

# A list of authors appears at the end of the paper.

Affiliations

1. Statistical Genetics Lab, QIMR Berghofer Medical Research Institute, Brisbane, Queensland, Australia.
2. School of Biomedical Sciences, Faculty of Health, Queensland University of Technology, Brisbane, Queensland, Australia
3. Center for Genomics and Personalised Health, Queensland University of Technology, Brisbane, Queensland, Australia
4. 23andMe, Inc, Sunnyvale, CA, USA.
5. Cancer Control Group, QIMR Berghofer Medical Research Institute, Brisbane, Queensland, Australia.
6. Faculty of Medicine, University of Queensland, Brisbane, Queensland, Australia.

*corresponding author- Mathias Seviiri, Mathias.Seviiri@qimrberghofer.edu.au

+61738453809, Statistical Genetics Lab, QIMR Berghofer Medical Research Institute, 300 Herston Road, Herston QLD 4006, Australia.

SUPPLEMENTARY METHODS

The UK Biobank and the respective GWAS

The UK Biobank is a population-based cohort comprising half a million adult participants (40-69 years) recruited between 2006 and 2010 from the United Kingdom. More information on participant recruitment, phenotype measurements, genotyping and participant follow up have been published elsewhere [1,2] and summarised here. Individuals were genotyped using the UK BiLEVE Axiom and UKB Axiom Arrays, and genetic data were imputed to the Haplotype Reference Consortium (HRC) and UK10K reference panels after genetic quality control procedures[2]. The study was approved by the National North West Multi-Centre Research Ethics Committee (ref. 11/NW/0382) in the United Kingdom and all participants provided written informed consent. More information about ethics oversight in the UK Biobank can be accessed at https://www.ukbiobank.ac.uk/ethics/.

In the UKB, BCC and SCC cases were ascertained through linkage of participant hospital data with cancer registries in the UK. In this present study, we selected histo-clinically confirmed BCC and SCC cases based on International Classification of Diseases (ICD) 10 codes (UKB data field 40006) or ICD 9 codes (data field 40013) and histological confirmation (data field 40011) based on the ICD for Oncology, 3rd edition codes for SCC (8070, 8071, 8072, 8073, 8074, 8075, 8076, 8078) and BCC (8090, 8091, 8092, 8093, 8094, 8097, 8098). We excluded cancer *in-situ* and self-reported cases for SCC and BCC. Controls had no history of any cancer diagnosis. We conducted the case-control GWAS for BCC (20,791 cases and 286,893 controls) and SCC (7,402 SCC cases and 286,892 controls) as described following in the Methods.

In addition, we conducted GWAS on pigmentation related traits in the UKB using the following phenotypes; facial aging (data field 1757), skin colour (data field 1717), childhood sunburns (data field 1737) and hair colour excluding red hair (data field 1747). We fitted linear mixed models using BOLT-LMM v2.3 [3] adjusting for age, age^2, sex and the interaction between them, and 10

PCs. For each phenotype we arranged its categorical values/codes in the exact order described in **Supplementary Data 2** and **Supplementary Data 3** then we applied rank normalisation (for each phenotype) prior to running linear models. For example, skin colour was ordered as; very fair, fair, light olive, dark olive, and brown while hair colour was; blonde, light brown, dark brown, and black. Red hair (data field 1747) was split off into a binary phenotype (red vs all others) and we conducted a case-control GWAS (14,354 cases, and 417,860 controls).

Using linear mixed modelling, we conducted quantitative GWAS on the following recently released blood biomarkers in the UKB; alanine aminotransferase (data field 30620), albumin (data field 30600), alkaline phosphatase (data field 30610), apolipoprotein A (data field 30630), apolipoprotein B (data field 30640), aspartate aminotransferase (data field 30650), C-reactive protein (data field 30710), calcium (data field 30680), cholesterol (data field 30690), creatinine (data field 30700), cystatin C (data field 30720), direct bilirubin (data field 30660), gamma glutamyltransferase (data field 30730), glucose (data field 30740), glycated haemoglobin (HbA1c) (data field 30750), High-density lipoprotein (HDL) cholesterol (data field 30760), insulin-like growth factor 1 (IGF-1) (data field 30770), low-density lipoprotein (LDL) direct (data field 30780), lipoprotein A (data field 30790), oestradiol (data field 30800), phosphate (data field 30810), sex hormone-binding globulin (SHBG) (data field 30830), testosterone (data field 30850), total bilirubin (data field 30840), total protein (data field 30860), triglycerides (data field 30870), urate (data field 30880), urea (data field 30670), and Vitamin D (data field 30890). We adjusted for age, sex and the first ten principal components. We included only participants of European ancestry and BOLT-LMM v2.3 [3] was used for the analysis. Sample sizes and details of each phenotype measurement are presented in **Supplementary Data 2**, and **Supplementary Data 3**. SNPs with MAF > 1% and imputation score greater than 0.3 were retained for subsequent analyses (Methods).

The QSkin Sun and Health Study (QSkin) and the respective GWAS

The QSkin Sun and Health Study (QSkin) has been extensively described elsewhere [4], and summarised here. Briefly, it is an Australian population based prospective cohort comprising over 43,000 adult participants aged 40-60 years recruited in 2011 with both clinical and skin-related trait data [4]. Participants residing in the state of Queensland were randomly sampled from the electoral rolls. In 2017 about 17,000 participants were genotyped using Illumina GSA arrays and imputed to the Haplotype Reference Consortium (HRC) panel version r1.1 2016 [5] using the Michigan Imputation Server [6]**.** Prior to imputation, genotype quality checks were conducted to remove individuals with high genotype missingness (>3%), relatedness (pihat >0.1875) and ancestry divergent from a European reference panel (> 6 SD on PC1 or 2 from European 1000 genome samples). In addition, SNPs with a MAF < 1% and Hardy-Weinberg equilibrium (HWE) p-value < 1×10^{-6} were excluded. After imputation, only SNPs with imputation quality score > 0.3 were retained. QSkin was approved by the Human Research Ethics Committee at QIMR Berghofer Medical Research Institute, Brisbane, Australia. All study participants provided written informed consent.

Data on skin and hair pigmentation traits, and skin cancers including KC were collected. KC (BCC and SCC) cases were ascertained through linkage of participant data with their health records in Medicare Australia and other Australian pathology registers [4]. Controls had no history of self-reported KC or actinic keratoses.

For ordinal traits we applied rank normalisation, and conducted GWAS using generalised linear models for mole count excluding melanoma cases (1=no moles, 2=some moles, 3=a few moles, and 4=many moles), skin burn type (1=not burn, 2=burn a little, 3=burn moderately, and 4=burn badly), skin colour (1=fair, 2=medium 3=olive/dark, and 4=black), skin tanning response (1=not tan, 2=tan lightly, 3=tan moderately, 4=tan deeply), hair colour excluding red (1=blonde, 2=light brown,

3=dark brown, and 4=black), and freckles (1=no freckles, 2=few freckles, 3=some freckles, and 4=many freckles). In addition, we conducted case-control GWAS for KC (8145 cases, 4797 controls), and red hair (973 cases, 15,202 controls). In all the GWAS above, we adjusted for age, age^2, sex and the interaction between them, and 10 PCs. PLINK2 (v2.00a2LM 31 March 2018 release) was used for the analysis [7]. Details on the sample size used for each GWAS are presented in **Supplementary Data 2** and **Supplementary Data 3**. As described in the **Methods** we tested the above phenotypes for genetic correlation with BCC and SCC and the results are presented in **Figure 1, Supplementary Data 1, Supplementary Data 2**, and only 22 phenotypes met the criteria to be included in the MTAG model for analysis.

The Electronic Medical Records and Genomics Network (eMERGE) and the KC GWAS

eMERGE is a research biorepository (N~19,000) in the USA comprising five electric medical databases; the Group Health Cooperative Biobank (by Group Health Cooperative), Personalised Medicine Research Project (by Marshfield Clinic Research Foundation), Vascular Diseases Biorepository (by Mayo Clinic), Nugene Project (by Northwestern University) and BioVu (by Vanderbilt University) [8,9]. Detailed description on participant enrollment, genotype data processing and the study design have been published before [8–12], and further information can be accessed through the database of Genotypes and Phenotypes (dbGaP, study accession: phs000360.v3.p1; https://www.ncbi.nlm.nih.gov/projects/gap/cgi-bin/study.cgi?study_id=phs000360.v3.p1). In our present study, 10,321 participants of European ancestry (1,565 KC cases and 8,756 KC free controls) were included in the KC GWAS. Cases were only considered if a participant reported a cancer code on two separate occasions when answering the survey questionnaire. Data on KC for was collected using ICD 9 codes and participants were genotyped using Illumina Human660W-Quad_v1_A array (San Diego, CA, USA) [8].

Genotype QC was performed to exclude SNPs with low call rate < 97%, MAF < 0.01 and significant deviation from HWE (P-value < 10^{-4} in controls and P-value < 10^{-10} in cases) [13]. Participants with > 3% missing genotypes were also excluded, and one of each pair of related individuals with PI_HAT scores > 0.2 [13]. Ancestry outliers with PC1 and PC2 values > 6 SD from the mean of the reference samples of Northern European ancestry (1000G British, CEU, and Finland) were also excluded [13]. PLINK 1.9 [7] was used for genotype cleaning. Next, the Michigan Imputation Server [6] and the HRC reference panel, version r1.1 2016 [5] were used for imputation. SNPs with an imputation quality score > 0.3 and MAF >1% were retained for the KC GWAS.

The Resource for Genetic Epidemiology Research on Aging (GERA) Cohort and the all-cancer GWAS

GERA cohort is a constituent study of Kaiser Permanente Research Program on Genes, Environment, and Health (RPGEH) based in the USA. It comprises about 78,000 participants with both genotype and phenotype data called using The International Classification of Diseases, Ninth Revision, Clinical Modification (ICD-9-CM) codes. Further details on RPGEH and GERA are elaborated on the database of Genotypes and Phenotypes (dbGaP, phs000674.v3.p3, https://www.ncbi.nlm.nih.gov/projects/gap/cgi-bin/study.cgi?study_id=phs000674.v3.p3). In particular, participants were genotyped using the Affymetrix Axiom arrays [14]. In our analysis cases for all-cancer were identified with ICD-9-CM codes for any cancer while controls had no history of any cancer, and data were accessed through dbGaP (study accession: phs000674.v3.p3, https://www.ncbi.nlm.nih.gov/projects/gap/cgi-bin/study.cgi?study_id=phs000674.v3.p3). Cases were only considered if a participant reported a cancer code on two separate occasions when answering the survey questionnaire. After performing genotype quality procedures, we retained 61,662 self-report European ancestry participants with low genotype missingness (< 3%) and minimal ancestry divergence European ancestry (> 6 sd of PC 1 or PC2 from the HapMap phase 3

CEU population) [15]. We also retained SNPs with MAF of >1%, call rate >95% and HWE P-value > 1×10-6. PLINK 1.9 [7] was used for genotype cleaning. Next we used the Michigan Imputation Server [6] and the HRC reference panel, version r1.1 2016 [5] for imputation. SNPs with an imputation quality score > 0.3 and MAF >1% were retained for association analysis.

Next, we used SAIGE [16] to conducted a case-control GWAS on all-cancer on 61,662 participants of European ancestry (18,621 cases and 43,041 controls), and adjusted for sex and 10 PCs. The subsequent GWAS were explored for genetic correlation with BCC and for MTAG analysis as described before (Methods).

Publicly accessed GWAS

Cutaneous Melanoma

We accessed and utilised summary statistics for the largest and most recently published CM GWAS meta-analysis, that included cohorts from Australia, Europe and America with participants of European descent [17]. The summary statistics were accessed from dbGap (accession study code: phs001868.v1.p1, https://www.ncbi.nlm.nih.gov/projects/gap/cgi-bin/study.cgi?study_id=phs001868.v1.p1). Detailed information of the GWAS meta-analysis including; the included cohorts, quality control metrics and other statistical analyses are published elsewhere [17], and summarised here. In brief, the meta-analysis was restricted to SNPs with MAF > 0.5% and imputation quality score > 0.5. For our study, we used GWAS summary data for 81,415 participants of European ancestry from 21 cohorts that included 30,134 histo-clinically confirmed CM cases (**Supplementary Table 2**). Thus, 23andMe participants as well as the UKB self-reported CM participants were excluded.

Hypothyroidism, type 1 diabetes, rheumatoid arthritis, and vitiligo

In order to assess the auto-immune traits hypothyroidism, type 1 diabetes and rheumatoid arthritis we accessed their respective publicly available data at ftp://share.sph.umich.edu/UKBB_SAIGE_HRC/. Details on how each GWAS was conducted are published elsewhere [16]. In summary, each GWAS was conducted using the UKB resource and SAIGE software [16] on participants of European ancestry. In addition, GWAS summary statistics on vitiligo [18] were utilised. The sample size and number of cases (where applicable) for each trait is presented in **Supplementary Data 1** and **Supplementary Data 2**.

Educational attainment, smoking, and body mass index

Using publicly available GWAS data, we also accessed and utilised summary statistics for educational attainment (accessible through http://ssgac.org/Data.php) [19], smoking (cigarettes per day)[20] which was accessed from the GWAS & Sequencing Consortium of Alcohol and Nicotine (GSCAN) Consortium https://genome.psych.umn.edu/index.php/GSCAN), and body mass index [21]. Details on the sample size are summarised in **Supplementary Data 1** and **Supplementary Data 2**. All GWAS were conducted in participants of European ancestry and excluded data from 23andMe.

SUPPLEMENTARY FIGURES

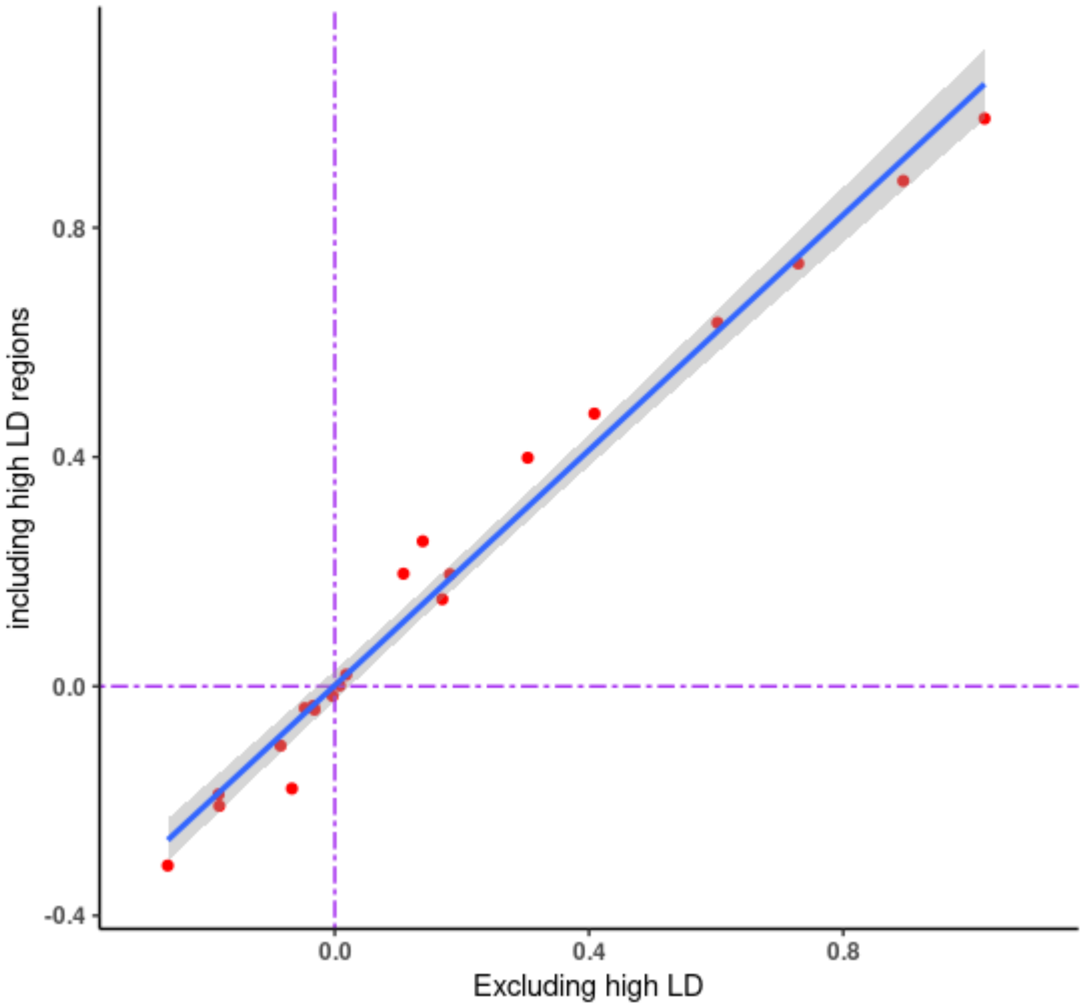

Supplementary Figure 1: Genetic correlation between basal cell carcinoma and 21 related traits after excluding genomic regions with very large effect sizes including; *HLA, ASIP, IRF4, MC1R, SLC45A2*, and *CDKN2A*. LD - linkage disequilibrium. The figure shows a high concordance (Pearson's correlation =0.99, 95% CI =0.98 - 1.00, P < 2.2 x 10^{-16}, two-tailed test) between the genetic correlation when the largest effect size regions are excluded, and when they are included. The shaded areas represent 95% confidence intervals for the line of best fit. Source data are provided as a Source Data file.

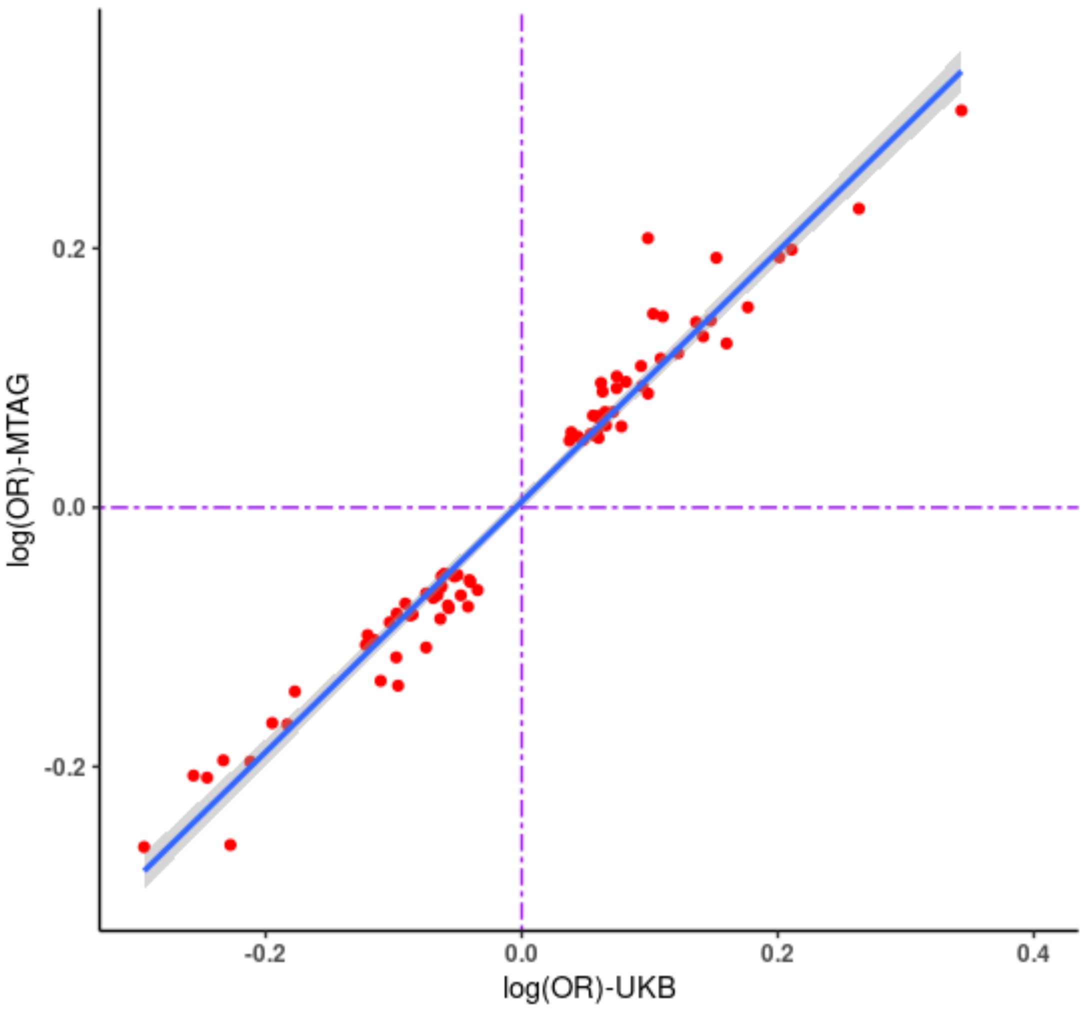

Supplementary Figure 2: Plot for the effect estimates (log odds ratio) for BCC MTAG versus BCC UKB GWAS excluding genomic regions with very large effect sizes including; *HLA, ASIP, IRF4, MC1R, SLC45A2*, and *CDKN2A*. OR - odds ratio, GWAS - Genome wide association study, UKB - United Kingdom Biobank, MTAG - multi-trait GWAS. BCC - Basal cell carcinoma. The figure shows a high concordance (Pearson's correlation =0.98, 95% CI =0.97 - 0.98, $P < 2.2 \times 10^{-16}$, two-tailed test) between the MTAG results and when the largest effect size regions are excluded. Shaded areas represent 95% confidence intervals for the line of best fit. Source data are provided as a Source Data file.

SUPPLEMENTARY TABLES

Supplementary Table 1: Gene-set pathways for BCC and SCC susceptibility

Trait	Pathway	Type	No. Genes	log (OR)	SE	P
BCC	pid_nfat_tfpathway	Curated	42	0.75	0.154	6.09E-07
	pid_il2_pi3k_pathway	Curated	33	0.76	0.16	9.67E-07
	nikolsky_breast_cancer_16q24_amplicon	Curated	49	2.62	0.25	7.37E-26
	nikolsky_breast_cancer_20q11_amplicon	Curated	31	2.45	0.296	7.57E-17
	martinez_response_to_trabectedin	Curated	48	0.79	0.168	1.29E-06
	reactome_melanin_biosynthesis	Curated	5	5.02	0.535	3.75E-21
	go_negative_regulation_of_regulatory_t_cell_differentiation	GO BP	3	3.43	0.689	3.19E-07
	go_melanosome_membrane	GO CC	13	1.77	0.315	9.59E-09
	go_mannosyl_oligosaccharide_1_2_alpha_mannosidase_activity	GO MF	6	2.24	0.483	1.74E-06
SCC	nikolsky_breast_cancer_16q24_amplicon	Curated	49	2.53	0.252	7.70E-24
	nikolsky_breast_cancer_20q11_amplicon	Curated	31	2.46	0.299	1.12E-16
	reactome_melanin_biosynthesis	Curated	5	5.41	0.539	7.13E-24
	go_melanin_biosynthetic_process	GO BP	19	1.23	0.237	1.05E-07
	go_melanosome_membrane	GO CC	13	1.83	0.318	4.46E-09
	go_mhc_class_ii_receptor_activity	GO MF	7	2.02	0.443	2.64E-06

GO- Gene ontology, BP- Biological Process, CC- Cellular Component, MF- Molecular Function

BCC - basal cell carcinoma SCC - squamous cell carcinoma

OR - odds ratio SE -standard error P - P-value

No. number of

Supplementary Table 2: Cohorts included in the cutaneous melanoma GWAS ***

Population	Study	Total N	Cases	Controls
European	GenoMEL Phase 1	3,238	1,075	2,163
European	GenoMEL Phase 2	2,053	925	1,128
United States of America	MDACC	2,942	1,924	1,018
Australian	AMFS	962	535	427
Australian	Q-MEGA_610k	4,689	912	3,777
Australian	Q-MEGA_omni	1,194	656	538
United Kingdom	GSEdinCIDRulcer	10,108	4,328	5,780
Australian	WAMHS	3,214	1,237	1,977
German	Essen-Heidelberg	2,404	1,189	1,215
French	MELARISK	1,326	511	815
United States of America	Harvard	3,330	410	2,920
United States of America	NCI_CPSII+PLCO+Rose	2,855	171	2,684
United Kingdom	UK Biobank confirmed	17,495	3,499	13,996
European-derived	MIA_PAH	4,774	1,933	2,841
Australian	EPIGENE	1,683	773	910
Australian	QSKIN	3,778	1,285	2,493
Greek	Greek	2,473	1,194	1,279
Italian	Italy	4,868	1,726	3,142
Spanish	Spain	6,923	3,523	3,400
United States of America	Michigan	27,409	1,198	26,211
European-derived	BNMS	3,831	1,130	2,701
	**Total**	**111,549**	**30,134**	**81,415**

GWAS *** conducted by Landi et al Nat Genetics 2020 and used as Trait 3 in our MTAG model. Please check it for further details

SUPPLEMENTARY NOTE ON DESCRIPTION OF THE PREVIOUSLY UNKNOWN LOCI

In this election we exclusively describe in detail the previously unknown loci identified in our study. These are implicated in KC initiation, pigmentation, cardiometabolic and immuno-regulatory pathways, as detailed below. Some loci are also noted to have pleiotropic effects, where they may influence more than one biological pathway for KC susceptibility.

KC development and progression

We identified seven loci with a potential role in the development and progression of KC; rs10766301 (*SOX6*) for BCC, rs10141120 *(MARK3*), rs10899466 (*GAB2*), rs142004400 (*CDKL1*) and rs472385 (*FRMD5)* for SCC, rs35563099/rs7098111 (*RAB11FIP2*), rs10876864 (*SUOX*) and rs12142181 (*EDN2*) for both BCC and SCC.

rs10141120 in *MARK3*; *MARK3* is a cell cycle regulator involved in the DNA damage response (e.g. following radiotherapy or treatment with alkylating agents) [22], and implicated in carcinogenesis e.g. for hepatocellular carcinoma [23]. In addition, rs10141120 is in LD with rs3825566 (r^2=0.94) and rs55859054 (r^2=0.96) which are lead SNPs for hair colour [24,25].

We also identified two variants for BCC and SCC; rs35563099 and rs7098111 respectively, near *RAB11FIP2* that were in high LD (r^2=0.98). *RAB11FIP2* is overexpressed in colorectal and gastric cancer cells where it facilitates their migration leading to cancer metastases [26,27]. It is likely that *RAB11FIP2* promotes keratinocyte cancer progression. rs35563099 is a lead SNP for skin low tanning response [28] and in LD with rs11198112 (r^2=0.99) for sunburns[24]. rs7098111 also is in LD with SNPs for skin/hair colour (rs11198112, r^2=0.57), freckles (rs10444039, r^2=0.99) and sunburns (rs11198112, r^2=0.98)[24,29,30].

rs10899466 in *GAB2* is in LD with rs10899501 for hair colour (r^2=0.80) [24,31]. Following inflammatory stimuli (e.g. by cytokines) *GAB2* is required in inflammatory signalling during tumorigenesis [32,33]. It enhances cancer cell proliferation e.g. in breast cancer [34,35]. Another SNP, rs10766301, is an intronic variant in *SOX6*. Although the direct role of *SOX6* in KC biology is unknown, it facilitates apoptosis in colorectal cancer, esophageal squamous cell carcinoma, pancreatic and ovarian cancer [36–39]. Conversely, its downregulation facilitates cancer progression [36,38,40]. rs10766301 is also in LD with rs2953060 (r^2=0.60) for sunburns [24].

rs142004400 in *CDKL1* promotes tumour cell proliferation, migration and invasion in melanoma, colorectal cancer, and breast cancer [41–43], whose downregulation facilitates apoptosis [41]. We also identified rs472385 in *FRMD5; FRMD5* modulates tumour progression by regulating cancer cell mobility and ROCK1-triggered kinase activity [44,45]. Its expression is downregulated in renal, breast and colorectal cancers [45]. rs472385 is also in LD with rs35654783 (r^2=0.76), a lead SNP for diastolic blood pressure [46]. Altered expression of *EDN2* (rs12142181) leads to tumourigenesis [47].

We also identified rs10876864 near *SUOX* (+1.78kb); expression of *SUOX* is associated with both proliferation and progression of oral squamous cell carcinoma, gastric cancer and hepatocellular carcinoma [48–50]. In addition, eQTL analysis showed that rs10876864 was strongly linked to expression of *SUOX* and *RPS26* in skin tissues (Table 1 and Table 2). *RPS26* regulates the tumour suppression activities of *p53* in response to DNA damage [51]. Thus, it is possible that rs10876864 is involved in proliferation and progression of keratinocyte cancers. However, it is also likely that rs10876864 might have pleiotropic effects on KC through immunomodulating pathways since it is also near *IKZF4* (-13.6kb); *IKZF4* is required to suppress/maintain FOXP3+ regulatory T cells [52,53], important in auto-immunity and self-recognition. It is in LD with lead SNPs for auto-immune traits e.g. T1D, and allergic disease, rheumatoid arthritis (in LD with rs773125, r^2=0.83) [54–56], immune-

suppressive medication use e.g. glucocorticoids (in LD with rs1689510, r^2=0.72), thyroid preparations (in LD with rs7302200, r^2= 0.71) and anti-asthmatic adrenergics inhalant use (in LD with rs34415530, r^2=0.72) [57].

Immune regulation pathway

Previous studies have reported a relationship between immune response and skin cancers [58], which has been key in developing immunotherapy (anti-PD1 and anti-CTLA4) for their treatment [59]. A number of loci suggest links to immune regulatory processes; rs2373232 (*CCRL2*), rs2111485 (*FAP*), rs10774625 (*ATXN2),* rs706779 (*IL2RA*), rs11059675 (*LRRC43* or *IL31) for BCC and* rs17391694 near (*GIPC2*) for SCC. rs2373232 is an intergenic variant between *CCRL2* (-4.337kb) and *CCR5* (+26.69kb) in high linkage disequilibrium (LD) r^2=1, with rs1015164 (in *CCR5*), a known SNP for HIV-1 viral load variation [60,61]. *CCR5* is generally involved in coordination of the immune response [62], and specifically regulates HIV-1 viral load and progression [63,64]. *CCRL2* is involved in regulating immune responses induced by chemokines [65].

rs2111485 is an intergenic variant between *FAP* (+10.49kb) and *IFIH1* (-13.05kb) and in high LD (r^2=0.89) with a nonsense SNP rs1990760 in *IFIH1; IFIH1* is involved in innate (anti-viral) immune response (e.g. against coronaviruses), autoimmunity and autoinflammatory response [66–68]. In addition, *FAP* plays a pro-tumourigenic role in several cancers including breast, colorectal, gastric and oesophageal cancer [69], and also facilitates immunosuppression to enhance colorectal and gastric cancer progression [70,71].

rs10774625, an intronic variant in *ATXN2,* is a lead SNP for hypothyroidism susceptibility [72], and in high LD (r^2=0.9) with a missense SNP rs3184504 in *SH2B3*; *SH2B3* is involved in mediating immune cell stimulation and inflammatory signalling [73,74]. Another SNP rs4409785 near *FAM76B* (

-190.7kb) is an *eQTL* for *SESN3* (in eQTLGEN), which regulates senescence in T-cells to influence the immune response during aging [75,76]. rs4409785 is also associated with thyroid preparations (medication use), hypothyroidism, vitiligo and rheumatoid arthritis [24,57,77,78].

At 10p15.1 rs706779 is an intronic variant in the *IL2RA* gene and lies a strong transcriptionally active enhancer in immune cells [79]. *IL2RA* controls the immune response (tolerance) by modulating the function of regulatory T-cells [80]. rs706779 is a lead SNP for T1D, Crohn's disease, vitiligo and in LD with rs7090530 (r^2=0.73) for hypothyroidism and thyroid medication [18,24,54,57,81]. rs11059675 in *LRRC43*, and near *IL31* (+9.58kb) is a lead SNP for psoriasis [82], and is in LD with rs7968808 (r^2= 0.98), a lead SNP for eczema [24]. *IL31* induces and modulates skin allergic diseases [83]. rs17391694 near *GIPC2* (+20.51kb) is a lead SNP for Crohn's disease, an inflammatory autoimmune disorder [81], and lung cancer [84].

Cardiometabolic pathway

For both BCC and SCC, we also identified loci that have been previously reported as being associated with cardiometabolic biomarkers. The BCC loci for this pathway included; rs174570 in *FADS2*, rs10774625 in *ATXN2*, and rs1136165 near *CKB* in BMI, whilst for SCC they included; rs3768321 in *PABPC4*, and rs1260326 near *GCKR*.

rs174570 is an intronic variant in *FADS2* and 12.68kb away from *FADS1* and it is a lead SNP for higher LDL cholesterol, total cholesterol and triglycerides levels, and is in LD with lead SNPs for PUFA levels in people of European descent e.g. rs174547 (r^2=0.36), rs174577 (r^2=0.34), and rs174538 (r^2=0.42) [85,86]. *FADS1/2* genes are involved in the downstream metabolism of the plasma omega-6 and omega-3 PUFA resulting in oncogenic inflammatory biomarkers (prostaglandins E,

thromboxane A2, and leukotriene B) [87]. rs1260326 in *GCKR* is a lead SNP for triglycerides, total cholesterol and fasting plasma glucose [88,89]. Mutations in *GCKR* are known to be diabetogenic [90]. rs3768321 in *PABPC4* is a lead SNP for HDL cholesterol [88].

Some variants in the cardiometabolic pathway had pleiotropic effects with pigmentation and autoimmune traits. For example rs1136165 in *CKB*, a lead SNP for BMI [24] is in LD with rs55859054 (r^2=0.89) and rs3825566 (r^2=0.88) which are associated with hair colour [24,25]. rs10774625 in *ATXN2* is linked to a spectrum of cardiometabolic markers; diastolic and systolic blood pressure, CVD, coronary artery disease, and LDL cholesterol (**Supplementary Data 4**) [24,46,91,92], is also a lead SNP for immune regulatory phenotypes; hypothyroidism and T1D [72,93].

Pigmentation pathways

Pigmentation is a crucial pathway in the development of BCC, SCC and melanoma. Known pigmentation genes like *MC1R* and *IRF4* play an important role in the genetic susceptibility to skin cancers. Three new loci were associated with pigmentation traits; rs77758638 (*AP3M2*) for SCC, rs2924552 (*TPCN2*), rs9878566 (*LINC00886*) for both BCC and SCC.

rs2924552 near *TPCN2* (+31.3kb); rare mutations in *TPCN2* results in blond rather than brown hair among Icelanders and the Dutch [94]. rs9878566 in *LINC00886* is in perfect LD (r^2=1.00) with rs9818780 for sunburns [24]. rs6889986 in *GPR98* is in LD with lead SNPS for hair colour; rs60325490 (r^2=0.69) and rs6860111 (r^2=0.69) [24,25]. rs77758638 in *AP3M2* is in LD with rs113060680 (r^2=0.93) for hair colour and skin tanning response [25]. However, there were also a number of loci with a potential role in KC initiation and progression that had pleiotropic effects with pigmentation traits (as explained above) e.g. rs35563099 and rs7098111 near *RAB11FIP2.*

BCC and SCC loci previously known for cutaneous melanoma susceptibility

Some loci were previously known for CM; *ATM*, and *SOX6* for BCC, and *GPR98, and DSTYK* for both BCC and SCC. rs73008229 near *ATM* in LD (r^2 ~1) with rs1801516 a missense variant (i.e. L76I) in *ATM*; *ATM* has a cell cycle function in DNA damage response due to radiotoxicity after radiotherapy [95–97]. rs1801516 has previously been associated with cutaneous melanoma [98–100]. Several other BCC SNPs are in high LD with lead SNPs for cutaneous melanoma; rs2369633 near *DSTYK* is in LD with rs11240396 (r^2=0.73), rs6889986 near *GPR98* locus is in LD with rs10942621 (r^2=0.71), rs10766301 in *SOX6* is in LD with rs2054095 (r^2=0.65) [17].

SUPPLEMENTARY NOTE ON ACKNOWLEDGEMENTS

eMERGE

The dbgap data for **eMERGE** we used in this study included samples from the University of Washington, Mayo Clinic, Marshfield Clinic, the Northwestern University and Vanderbilt University. In the following paragraphs, we acknowledge each study separately:

Group Health Cooperative/University of Washington

Funding support for Alzheimer's Disease Patient Registry (ADPR) and Adult Changes in Thought (ACT) study was provided by a U01 from the National Institute on Aging (Eric B. Larson, PI, U01AG006781). A gift from the 3M Corporation was used to expand the ACT cohort. DNA aliquots sufficient for GWAS from ADPR probable AD cases, which had been enrolled in genetic differences in Alzheimer's cases and controls (Walter Kukull, PI, R01 AG007584) and obtained under that grant, were made available to eMERGE without charge. Funding support for genotyping, which was performed at Johns Hopkins University, was provided by the NIH (U01HG004438). Genome-wide association analyses were supported through a Cooperative Agreement from the National Human Genome Research Institute, U01HG004610 (Eric B. Larson, PI).

Mayo Clinic

Samples and associated genotype and phenotype data used in this study were provided by the Mayo Clinic. Funding support for the Mayo Clinic was provided through a cooperative agreement with the National Human Genome Research Institute (NHGRI), Grant #: UOIHG004599; and by grant HL75794 from the National Heart Lung and Blood Institute (NHLBI). Funding support for genotyping, which was performed at The Broad Institute, was provided by the NIH (U01HG004424).

Marshfield Clinic

Research Foundation Funding support for the Personalised Medicine Research Project (PMRP) was provided through a cooperative agreement (U01HG004608) with the National Human Genome Research Institute (NHGRI), with additional funding from the National Institute for General Medical Sciences (NIGMS). The samples used for PMRP analyses were obtained with funding from Marshfield Clinic, Health Resources Service Administration Office of Rural Health Policy grant number D1A RH00025, and Wisconsin Department of Commerce Technology Development Fund contract number TDF FYO10718. Funding support for genotyping, which was performed at Johns Hopkins University, was provided by the NIH (U01HG004438).

Northwestern University

Samples and data used in this study were provided by the NUgene Project (www.nugene.org). Funding support for the NUgene Project was provided by the Northwestern University's Center for Genetic Medicine, Northwestern University and Northwestern Memorial Hospital. Assistance with phenotype harmonisation was provided by the eMERGE Coordinating Center (Grant number U01HG04603). This study was funded through the NIH, NHGRI eMERGE Network (U01HG004609). Funding support for genotyping, which was performed at The Broad Institute, was provided by the NIH (U01HG004424).

Vanderbilt University

Funding support for the Vanderbilt Genome-Electronic Records (VGER) project was provided through a cooperative agreement (U01HG004603) with the National Human Genome Research Institute (NHGRI) with additional funding from the National Institute of General Medical Sciences (NIGMS). The dataset and samples used for the VGER analyses were obtained from Vanderbilt University Medical Center's BioVU, which is supported by institutional funding and by the

Vanderbilt CTSA grant UL1RR024975 from NCRR/NIH. Funding support for genotyping, which was performed at The Broad Institute, was provided by the NIH (U01HG004424).

Assistance with phenotype harmonisation and genotype data cleaning was provided by the eMERGE Administrative Coordinating Center (U01HG004603) and the National Center for Biotechnology Information (NCBI). The datasets used for the analyses described in this manuscript were obtained from dbGaP at http://www.ncbi.nlm.nih.gov/gap through dbGaP accession number phs000360.v3.p1, https://www.ncbi.nlm.nih.gov/projects/gap/cgi-bin/study.cgi?study_id=phs000360.v3.p1.

The 23andMe Research Team

Stella Aslibekyan, Adam Auton, Elizabeth Babalola, Robert K. Bell, Jessica Bielenberg, Katarzyna Bryc, Emily Bullis, Daniella Coker, Gabriel Cuellar Partida, Devika Dhamija, Sayantan Das, Sarah L. Elson, Teresa Filshtein, Kipper Fletez-Brant, Pierre Fontanillas, Will Freyman, Pooja M. Gandhi, Karl Heilbron, Barry Hicks, David A. Hinds, Ethan M. Jewett, Yunxuan Jiang, Katelyn Kukar, Keng-Han Lin, Maya Lowe, Jey McCreight, Matthew H. McIntyre, Steven J. Micheletti, Meghan E. Moreno, Joanna L. Mountain, Priyanka Nandakumar, Elizabeth S. Noblin, Jared O'Connell, Aaron A. Petrakovitz, G. David Poznik, Morgan Schumacher, Anjali J. Shastri, Janie F. Shelton, Jingchunzi Shi, Suyash Shringarpure, Vinh Tran, Joyce Y. Tung, Xin Wang, Wei Wang, Catherine H. Weldon, Peter Wilton, Alejandro Hernandez, Corinna Wong, and Christophe Toukam Tchakouté

SUPPLEMENTARY REFERENCES

1. Sudlow, C. *et al.* UK biobank: an open access resource for identifying the causes of a wide range of complex diseases of middle and old age. *PLoS Med.* **12**, e1001779 (2015).
2. Bycroft, C. *et al.* The UK Biobank resource with deep phenotyping and genomic data. *Nature* **562**, 203–209 (2018).
3. Loh, P.-R. *et al.* Efficient Bayesian mixed-model analysis increases association power in large cohorts. *Nat. Genet.* **47**, 284–290 (2015).
4. Olsen, C. M. *et al.* Cohort profile: The QSkin Sun and Health Study. *Int. J. Epidemiol.* **41**, 929–929i (2012).
5. McCarthy, S. *et al.* A reference panel of 64,976 haplotypes for genotype imputation. *Nat. Genet.* **48**, 1279–1283 (2016).
6. Das, S. *et al.* Next-generation genotype imputation service and methods. *Nat. Genet.* **48**, 1284–1287 (2016).
7. Chang, C. C. *et al.* Second-generation PLINK: rising to the challenge of larger and richer datasets. *Gigascience* **4**, 7 (2015).
8. McCarty, C. A. *et al.* The eMERGE Network: a consortium of biorepositories linked to electronic medical records data for conducting genomic studies. *BMC Med. Genomics* **4**, 13 (2011).
9. Kho, A. N. *et al.* Electronic medical records for genetic research: results of the eMERGE consortium. *Sci. Transl. Med.* **3**, 79re1 (2011).
10. McCarty, C. A., Peissig, P., Caldwell, M. D. & Wilke, R. A. The Marshfield Clinic Personalized Medicine Research Project: 2008 scientific update and lessons learned in the first 6 years. *Per. Med.* **5**, 529–542 (2008).
11. Kukull, W. A. *et al.* Dementia and Alzheimer Disease Incidence. *Archives of Neurology* vol. 59 1737 (2002).
12. Roden, D. M. *et al.* Development of a large-scale de-identified DNA biobank to enable personalized medicine. *Clin. Pharmacol. Ther.* **84**, 362–369 (2008).
13. Liyanage, U. E. *et al.* Combined analysis of keratinocyte cancers identifies novel genome-wide loci. *Hum. Mol. Genet.* **28**, 3148–3160 (2019).

14. Banda, Y. *et al.* Characterizing Race/Ethnicity and Genetic Ancestry for 100,000 Subjects in the Genetic Epidemiology Research on Adult Health and Aging (GERA) Cohort. *Genetics* **200**, 1285–1295 (2015).
15. International HapMap Consortium. The International HapMap Project. *Nature* **426**, 789–796 (2003).
16. Zhou, W. *et al.* Efficiently controlling for case-control imbalance and sample relatedness in large-scale genetic association studies. *Nat. Genet.* **50**, 1335–1341 (2018).
17. Landi, M. T. *et al.* Genome-wide association meta-analyses combining multiple risk phenotypes provide insights into the genetic architecture of cutaneous melanoma susceptibility. *Nat. Genet.* **52**, 494–504 (2020).
18. Jin, Y. *et al.* Genome-wide association studies of autoimmune vitiligo identify 23 new risk loci and highlight key pathways and regulatory variants. *Nat. Genet.* **48**, 1418–1424 (2016).
19. Okbay, A. *et al.* Genome-wide association study identifies 74 loci associated with educational attainment. *Nature* **533**, 539–542 (2016).
20. Liu, M. *et al.* Association studies of up to 1.2 million individuals yield new insights into the genetic etiology of tobacco and alcohol use. *Nat. Genet.* **51**, 237–244 (2019).
21. Yengo, L. *et al.* Meta-analysis of genome-wide association studies for height and body mass index in ∼700000 individuals of European ancestry. *Hum. Mol. Genet.* **27**, 3641–3649 (2018).
22. Owusu, M. *et al.* Mapping the Human Kinome in Response to DNA Damage. *Cell Rep.* **26**, 555–563.e6 (2019).
23. Kato, T. *et al.* Isolation of a novel human gene, MARKL1, homologous to MARK3 and its involvement in hepatocellular carcinogenesis. *Neoplasia* **3**, 4–9 (2001).
24. Kichaev, G. *et al.* Leveraging Polygenic Functional Enrichment to Improve GWAS Power. *Am. J. Hum. Genet.* **104**, 65–75 (2019).
25. Morgan, M. D. *et al.* Genome-wide study of hair colour in UK Biobank explains most of the SNP heritability. *Nat. Commun.* **9**, 5271 (2018).
26. Dong, W., Qin, G. & Shen, R. Rab11-FIP2 promotes the metastasis of gastric cancer cells. *International journal of cancer* **138**, (2016).
27. Dong, W. & Wu, X. Overexpression of Rab11-FIP2 in colorectal cancer cells promotes tumor migration and angiogenesis through increasing secretion of PAI-1. *Cancer Cell Int.* **18**, 35 (2018).

28. Visconti, A. *et al.* Genome-wide association study in 176,678 Europeans reveals genetic loci for tanning response to sun exposure. *Nat. Commun.* **9**, 1684 (2018).

29. Adhikari, K. *et al.* A GWAS in Latin Americans highlights the convergent evolution of lighter skin pigmentation in Eurasia. *Nat. Commun.* **10**, 358 (2019).

30. Endo, C. *et al.* Genome-wide association study in Japanese females identifies fifteen novel skin-related trait associations. *Sci. Rep.* **8**, 8974 (2018).

31. Hysi, P. G. *et al.* Genome-wide association meta-analysis of individuals of European ancestry identifies new loci explaining a substantial fraction of hair color variation and heritability. *Nat. Genet.* **50**, 652–656 (2018).

32. Kondreddy, V., Magisetty, J., Keshava, S., Vijaya Mohan Rao, L. & Pendurthi, U. R. Gab2 (Grb2-Associated Binder2) Plays a Crucial Role in Inflammatory Signaling and Endothelial Dysfunction. *Arterioscler. Thromb. Vasc. Biol.* **41**, 1987 (2021).

33. Ding, C.-B., Yu, W.-N., Feng, J.-H. & Luo, J.-M. Structure and function of Gab2 and its role in cancer (Review). *Mol. Med. Rep.* **12**, 4007 (2015).

34. Bentires-Alj, M. *et al.* A role for the scaffolding adapter GAB2 in breast cancer. *Nat. Med.* **12**, 114–121 (2006).

35. Brummer, T. *et al.* Increased proliferation and altered growth factor dependence of human mammary epithelial cells overexpressing the Gab2 docking protein. *J. Biol. Chem.* **281**, 626–637 (2006).

36. Qin, Y.-R. *et al.* Characterization of tumor-suppressive function of SOX6 in human esophageal squamous cell carcinoma. *Clin. Cancer Res.* **17**, 46–55 (2011).

37. Jiang, W. *et al.* Identification of Sox6 as a regulator of pancreatic cancer development. *J. Cell. Mol. Med.* **22**, 1864–1872 (2018).

38. Li, Y., Xiao, M. & Guo, F. The role of Sox6 and Netrin-1 in ovarian cancer cell growth, invasiveness, and angiogenesis. *Tumour Biol.* **39**, 1010428317705508 (2017).

39. Li, Y.-C. *et al.* MicroRNA-766 targeting regulation of SOX6 expression promoted cell proliferation of human colorectal cancer. *Onco. Targets. Ther.* **8**, 2981–2988 (2015).

40. Guo, X., Yang, M., Gu, H., Zhao, J. & Zou, L. Decreased expression of SOX6 confers a poor prognosis in hepatocellular carcinoma. *Cancer Epidemiol.* **37**, 732–736 (2013).

41. Song, Z., Lin, J., Sun, Z., Ni, J. & Sha, Y. RNAi-mediated downregulation of CDKL1 inhibits growth

and colony-formation ability, promotes apoptosis of human melanoma cells. *J. Dermatol. Sci.* **79**, (2015).

42. Qin, C. *et al.* CDKL1 promotes tumor proliferation and invasion in colorectal cancer. *Onco. Targets. Ther.* **10**, 1613–1624 (2017).
43. Tang, L., Gao, Y., Yan, F. & Tang, J. Evaluation of cyclin-dependent kinase-like 1 expression in breast cancer tissues and its regulation in cancer cell growth. *Cancer Biother. Radiopharm.* **27**, 392–398 (2012).
44. Hu, J. *et al.* FERM domain-containing protein FRMD5 regulates cell motility via binding to integrin β5 subunit and ROCK1. *FEBS Letters* vol. 588 4348–4356 (2014).
45. Wang, T. *et al.* FERM-containing protein FRMD5 is a p120-catenin interacting protein that regulates tumor progression. *FEBS Lett.* **586**, 3044–3050 (2012).
46. Hoffmann, T. J. *et al.* Genome-wide association analyses using electronic health records identify new loci influencing blood pressure variation. *Nat. Genet.* **49**, 54–64 (2017).
47. Khimji, A.-K. & Rockey, D. C. Endothelin--biology and disease. *Cell. Signal.* **22**, 1615–1625 (2010).
48. Nakamura, K. *et al.* SUOX is negatively associated with multistep carcinogenesis and proliferation in oral squamous cell carcinoma. *Med. Mol. Morphol.* **51**, 102–110 (2018).
49. Jin, G.-Z. *et al.* SUOX is a promising diagnostic and prognostic biomarker for hepatocellular carcinoma. *J. Hepatol.* **59**, 510–517 (2013).
50. Yano, Y. *et al.* Sulfite Oxidase Is a Novel Prognostic Biomarker of Advanced Gastric Cancer. *In Vivo* **35**, 229–237 (2021).
51. Cui, D. *et al.* The ribosomal protein S26 regulates p53 activity in response to DNA damage. *Oncogene* **33**, 2225–2235 (2014).
52. Sharma, M. D. *et al.* An inherently bifunctional subset of Foxp3+ T helper cells is controlled by the transcription factor eos. *Immunity* **38**, 998–1012 (2013).
53. Pan, F. *et al.* Eos mediates Foxp3-dependent gene silencing in CD4+ regulatory T cells. *Science* **325**, 1142–1146 (2009).
54. Onengut-Gumuscu, S. *et al.* Fine mapping of type 1 diabetes susceptibility loci and evidence for colocalization of causal variants with lymphoid gene enhancers. *Nat. Genet.* **47**, 381–386 (2015).
55. Ferreira, M. A. *et al.* Shared genetic origin of asthma, hay fever and eczema elucidates allergic disease

biology. *Nat. Genet.* **49**, 1752–1757 (2017).

56. Laufer, V. A. *et al.* Genetic influences on susceptibility to rheumatoid arthritis in African-Americans. *Hum. Mol. Genet.* **28**, 858–874 (2019).

57. Wu, Y. *et al.* Genome-wide association study of medication-use and associated disease in the UK Biobank. *Nat. Commun.* **10**, 1891 (2019).

58. Chahal, H. S. *et al.* Genome-wide association study identifies 14 novel risk alleles associated with basal cell carcinoma. *Nat. Commun.* **7**, 12510 (2016).

59. Rotte, A. Combination of CTLA-4 and PD-1 blockers for treatment of cancer. *J. Exp. Clin. Cancer Res.* **38**, 255 (2019).

60. McLaren, P. J. *et al.* Polymorphisms of large effect explain the majority of the host genetic contribution to variation of HIV-1 virus load. *Proc. Natl. Acad. Sci. U. S. A.* **112**, 14658–14663 (2015).

61. Ekenberg, C. *et al.* Association Between Single-Nucleotide Polymorphisms in HLA Alleles and Human Immunodeficiency Virus Type 1 Viral Load in Demographically Diverse, Antiretroviral Therapy-Naive Participants From the Strategic Timing of AntiRetroviral Treatment Trial. *J. Infect. Dis.* **220**, 1325–1334 (2019).

62. Hüttenrauch, F., Pollok-Kopp, B. & Oppermann, M. G protein-coupled receptor kinases promote phosphorylation and beta-arrestin-mediated internalization of CCR5 homo- and hetero-oligomers. *J. Biol. Chem.* **280**, 37503–37515 (2005).

63. Alvarez, V., López-Larrea, C. & Coto, E. Mutational analysis of the CCR5 and CXCR4 genes (HIV-1 co-receptors) in resistance to HIV-1 infection and AIDS development among intravenous drug users. *Hum. Genet.* **102**, 483–486 (1998).

64. Carrington, M., Dean, M., Martin, M. P. & O'Brien, S. J. Genetics of HIV-1 infection: chemokine receptor CCR5 polymorphism and its consequences. *Hum. Mol. Genet.* **8**, 1939–1945 (1999).

65. Hartmann, T. N. *et al.* Human B cells express the orphan chemokine receptor CRAM-A/B in a maturation-stage-dependent and CCL5-modulated manner. *Immunology* vol. 125 252–262 (2008).

66. Kato, H. *et al.* Differential roles of MDA5 and RIG-I helicases in the recognition of RNA viruses. *Nature* **441**, 101–105 (2006).

67. Wawrusiewicz-Kurylonek, N. *et al.* The interferon-induced helicase C domain-containing protein 1 gene variant (rs1990760) as an autoimmune-based pathology susceptibility factor. *Immunobiology* **225**,

151864 (2020).

68. Dias Junior, A. G., Sampaio, N. G. & Rehwinkel, J. A Balancing Act: MDA5 in Antiviral Immunity and Autoinflammation. *Trends Microbiol.* **27**, 75–85 (2019).

69. Puré, E. & Blomberg, R. Pro-tumorigenic roles of fibroblast activation protein in cancer: back to the basics. *Oncogene* **37**, 4343–4357 (2018).

70. Chen, L., Qiu, X., Wang, X. & He, J. FAP positive fibroblasts induce immune checkpoint blockade resistance in colorectal cancer via promoting immunosuppression. *Biochem. Biophys. Res. Commun.* **487**, 8–14 (2017).

71. Wen, X. *et al.* Fibroblast Activation Protein-α-Positive Fibroblasts Promote Gastric Cancer Progression and Resistance to Immune Checkpoint Blockade. *Oncology Research Featuring Preclinical and Clinical Cancer Therapeutics* vol. 25 629–640 (2017).

72. Pickrell, J. K. *et al.* Detection and interpretation of shared genetic influences on 42 human traits. *Nat. Genet.* **48**, 709–717 (2016).

73. Devallière, J. & Charreau, B. The adaptor Lnk (SH2B3): an emerging regulator in vascular cells and a link between immune and inflammatory signaling. *Biochem. Pharmacol.* **82**, 1391–1402 (2011).

74. Katayama, H. *et al.* Lnk prevents inflammatory $CD8^+$ T-cell proliferation and contributes to intestinal homeostasis. *Eur. J. Immunol.* **44**, 1622–1632 (2014).

75. Pereira, B. I. *et al.* Sestrins induce natural killer function in senescent-like CD8 T cells. *Nat. Immunol.* **21**, 684–694 (2020).

76. Lanna, A. *et al.* A sestrin-dependent Erk-Jnk-p38 MAPK activation complex inhibits immunity during aging. *Nat. Immunol.* **18**, 354–363 (2017).

77. Okada, Y. *et al.* Genetics of rheumatoid arthritis contributes to biology and drug discovery. *Nature* **506**, 376–381 (2014).

78. Jin, Y. *et al.* Genome-wide association analyses identify 13 new susceptibility loci for generalized vitiligo. *Nat. Genet.* **44**, 676–680 (2012).

79. Ward, L. D. & Kellis, M. HaploReg: a resource for exploring chromatin states, conservation, and regulatory motif alterations within sets of genetically linked variants. *Nucleic Acids Res.* **40**, D930–4 (2012).

80. Goudy, K. *et al.* Human IL2RA null mutation mediates immunodeficiency with lymphoproliferation

and autoimmunity. *Clin. Immunol.* **146**, 248–261 (2013).

81. Liu, J. Z. *et al.* Association analyses identify 38 susceptibility loci for inflammatory bowel disease and highlight shared genetic risk across populations. *Nat. Genet.* **47**, 979–986 (2015).
82. Tsoi, L. C. *et al.* Large scale meta-analysis characterizes genetic architecture for common psoriasis associated variants. *Nat. Commun.* **8**, 15382 (2017).
83. Dillon, S. R. *et al.* Interleukin 31, a cytokine produced by activated T cells, induces dermatitis in mice. *Nat. Immunol.* **5**, 752–760 (2004).
84. McKay, J. D. *et al.* Large-scale association analysis identifies new lung cancer susceptibility loci and heterogeneity in genetic susceptibility across histological subtypes. *Nat. Genet.* **49**, 1126–1132 (2017).
85. Lemaitre, R. N. *et al.* Genetic loci associated with plasma phospholipid n-3 fatty acids: a meta-analysis of genome-wide association studies from the CHARGE Consortium. *PLoS Genet.* **7**, e1002193 (2011).
86. Guan, W. *et al.* Genome-wide association study of plasma N6 polyunsaturated fatty acids within the cohorts for heart and aging research in genomic epidemiology consortium. *Circ. Cardiovasc. Genet.* **7**, 321–331 (2014).
87. Azrad, M., Turgeon, C. & Demark-Wahnefried, W. Current evidence linking polyunsaturated Fatty acids with cancer risk and progression. *Front. Oncol.* **3**, 224 (2013).
88. Klarin, D. *et al.* Genetics of blood lipids among ~300,000 multi-ethnic participants of the Million Veteran Program. *Nat. Genet.* **50**, 1514–1523 (2018).
89. Hoffmann, T. J. *et al.* A large electronic-health-record-based genome-wide study of serum lipids. *Nat. Genet.* **50**, 401–413 (2018).
90. Warner, J. P., Leek, J. P., Intody, S., Markham, A. F. & Bonthron, D. T. Human glucokinase regulatory protein (GCKR): cDNA and genomic cloning, complete primary structure, and chromosomal localization. *Mamm. Genome* **6**, (1995).
91. van der Harst, P. & Verweij, N. Identification of 64 Novel Genetic Loci Provides an Expanded View on the Genetic Architecture of Coronary Artery Disease. *Circ. Res.* **122**, 433–443 (2018).
92. Sung, Y. J. *et al.* A Large-Scale Multi-ancestry Genome-wide Study Accounting for Smoking Behavior Identifies Multiple Significant Loci for Blood Pressure. *Am. J. Hum. Genet.* **102**, 375–400 (2018).
93. Plagnol, V. *et al.* Genome-wide association analysis of autoantibody positivity in type 1 diabetes cases. *PLoS Genet.* **7**, e1002216 (2011).

94. Sulem, P. *et al.* Two newly identified genetic determinants of pigmentation in Europeans. *Nat. Genet.* **40**, 835–837 (2008).

95. Kerns, S. L. *et al.* Radiogenomics Consortium Genome-Wide Association Study Meta-Analysis of Late Toxicity After Prostate Cancer Radiotherapy. *J. Natl. Cancer Inst.* **112**, 179–190 (2020).

96. Wu, X. *et al.* ATM phosphorylation of Nijmegen breakage syndrome protein is required in a DNA damage response. *Nature* vol. 405 477–482 (2000).

97. Bakkenist, C. J. & Kastan, M. B. DNA damage activates ATM through intermolecular autophosphorylation and dimer dissociation. *Nature* **421**, 499–506 (2003).

98. Duffy, D. L. *et al.* Novel pleiotropic risk loci for melanoma and nevus density implicate multiple biological pathways. *Nat. Commun.* **9**, 4774 (2018).

99. Barrett, J. H. *et al.* Genome-wide association study identifies three new melanoma susceptibility loci. *Nat. Genet.* **43**, 1108–1113 (2011).

100. Ransohoff, K. J. *et al.* Two-stage genome-wide association study identifies a novel susceptibility locus associated with melanoma. *Oncotarget* **8**, 17586–17592 (2017).
