# Supplementary material for: A multi-phenotype analysis reveals 19 susceptibility loci for basal cell carcinoma and 15 for squamous cell carcinoma
Source: Nat Commun. 2022 Dec 10;13:7650. doi: 10.1038/s41467-022-35345-8 (PMC9741635; doi:10.1038/s41467-022-35345-8)
Supplement: Supplementary file 3 — Description of Additional Supplementary Files [file 41467_2022_35345_MOESM3_ESM.pdf]

Description of Additional Supplementary Files

File Name: Supplementary Data 1
Description: Bivariate genetic correlation for the phenotypes included in MTAG model.

File Name: Supplementary Data 2
Description: Traits that were excluded from the MTAG analysis.

File Name: Supplementary Data 3
Description: The genetically correlated phenotypes that were included in the MTAG analysis.

File Name: Supplementary Data 4
Description: Basal cell carcinoma loci from the MTAG analysis.

File Name: Supplementary Data 5
Description: Squamous cell carcinoma loci from the MTAG analysis.

File Name: Supplementary Data 6
Description: Single nucleotide polymorphisms included in the polygenic risk score derived from basal cell carcinoma single trait GWAS.

File Name: Supplementary Data 7
Description: Single nucleotide polymorphisms included in the polygenic risk score derived from basal cell carcinoma MTAG analysis.

File Name: Supplementary Data 8
Description: Shared SNPs between the MTAG and the SINGLE BCC PRS based on the nearest gene.
