# Supplementary material for: A multi-phenotype analysis reveals 19 susceptibility loci for basal cell carcinoma and 15 for squamous cell carcinoma
Source: Nat Commun. 2022 Dec 10;13:7650. doi: 10.1038/s41467-022-35345-8 (PMC9741635; doi:10.1038/s41467-022-35345-8)
Supplement: Supplementary file 12 — Reporting Summary [file 41467_2022_35345_MOESM12_ESM.pdf]

nature portfolio

Corresponding author(s): Mathias Seviiri

Last updated by author(s): 23/11/22

Reporting Summary

Nature Portfolio wishes to improve the reproducibility of the work that we publish. This form provides structure for consistency and transparency in reporting. For further information on Nature Portfolio policies, see our Editorial Policies and the Editorial Policy Checklist.

Statistics

For all statistical analyses, confirm that the following items are present in the figure legend, table legend, main text, or Methods section.

n/a	Confirmed	
☐	☒	The exact sample size (n) for each experimental group/condition, given as a discrete number and unit of measurement
☒	☐	A statement on whether measurements were taken from distinct samples or whether the same sample was measured repeatedly
☐	☒	The statistical test(s) used AND whether they are one- or two-sided<br>*Only common tests should be described solely by name; describe more complex techniques in the Methods section.*
☐	☒	A description of all covariates tested
☐	☒	A description of any assumptions or corrections, such as tests of normality and adjustment for multiple comparisons
☐	☒	A full description of the statistical parameters including central tendency (e.g. means) or other basic estimates (e.g. regression coefficient) AND variation (e.g. standard deviation) or associated estimates of uncertainty (e.g. confidence intervals)
☐	☒	For null hypothesis testing, the test statistic (e.g. F, t, r) with confidence intervals, effect sizes, degrees of freedom and P value noted<br>*Give P values as exact values whenever suitable.*
☒	☐	For Bayesian analysis, information on the choice of priors and Markov chain Monte Carlo settings
☒	☐	For hierarchical and complex designs, identification of the appropriate level for tests and full reporting of outcomes
☐	☒	Estimates of effect sizes (e.g. Cohen's d, Pearson's r), indicating how they were calculated

Our web collection on statistics for biologists contains articles on many of the points above.

Software and code

Policy information about availability of computer code

Data collection	Data for the two traits of interest (BCC and SCC) were collected by the UK Biobank team using online questionnaires and through data linkage of participant with National Cancer Registers. Detailed description is found here https://biobank.ndph.ox.ac.uk/showcase/field.cgi?id=40006, and https://www.ncbi.nlm.nih.gov/pmc/articles/PMC4380465/
Data analysis	The following software were used for data analysis:PLINK2 (v2.00a2LM 31 March 2018 release), PLINK2 (v2.00a3LM 5 May 2021 release), R version 4.0.2, R package PredictABEL version 1.2-4, MAGMA v.1.7, FUMA v.1.3.6 , LDSC version 1.0.1, MTAG software version 1.0.8, SAIGE software v 0.38. The code used to generate the key results in this study can be freely accessed at https://github.com/mathiasS-hub/KC_MTAG_NatComm_Code.

For manuscripts utilizing custom algorithms or software that are central to the research but not yet described in published literature, software must be made available to editors and reviewers. We strongly encourage code deposition in a community repository (e.g. GitHub). See the Nature Portfolio guidelines for submitting code & software for further information.

Data

Policy information about availability of data

All manuscripts must include a data availability statement. This statement should provide the following information, where applicable:

- Accession codes, unique identifiers, or web links for publicly available datasets
- A description of any restrictions on data availability
- For clinical datasets or third party data, please ensure that the statement adheres to our policy

The full GWAS summary statistics generated in this study have been deposited in the NHGRI-EBI GWAS Catalog under accession code GCST90137411 (https://www.ebi.ac.uk/gwas/studies/GCST90137411) for BCC and GCST90137412 (https://www.ebi.ac.uk/gwas/studies/GCST90137412) for SCC. The PRS generated in this paper are provided with this paper in Supplementary Data 6 and 7. The BCC and SCC independent genome-wide significant SNPs (both for the discovery MTAG results and replication from 23andMe) generated in this study are provided with this paper in the Supplementary Data 4, and 5 files respectively. Source data for the figures are provided with this paper in the Source Data File. Genotype and phenotype data for the UK Biobank are available through application via https://www.ukbiobank.ac.uk/, for the Canadian Longitudinal Study on Aging (CLSA) at www.clsa-elcv.ca , and for QSkin through application to Prof. David Whiteman (David.Whiteman@qimrberghofer.edu.au), the principal investigator. Data from the Resource for Genetic Epidemiology Research on Aging (GERA) Cohort (dbGap accession phs000674.v3.p3, https://www.ncbi.nlm.nih.gov/projects/gap/cgi-bin/study.cgi?study_id=phs000674.v3.p3), and the Electronic Medical Records and Genomics Network (eMERGE) (dbGaP, study accession: phs000360.v3.p1, https://www.ncbi.nlm.nih.gov/projects/gap/cgi-bin/study.cgi?study_id=phs000360.v3.p1) can be accessed from dpGAP. The cutaneous melanoma GWAS summary statistics used in this paper were were from Landi et al 2020 20, are publicly available from dbGap (accession study code: phs001868.v1.p1, https://www.ncbi.nlm.nih.gov/projects/gap/cgi-bin/study.cgi?study_id=phs001868.v1.p1). The following trait-specific GWAS summary statistics were also used in this paper and are publicly available through the following consortia or resources; educational attainment by Okbay et al 2016 27 (downloadable from the SSGAC website http://ssgac.org/Data.php), smoking (cigarettes per day) by Liu et al 2019 28 (downloadable from the GSCAN Consortium website https://genome.psych.umn.edu/index.php/GSCAN), and auto-immune traits from by Zhou et al 2018 25 (available at ftp://share.sph.umich.edu/UKBB_SAIGE_HRC/).

Human research participants

Policy information about studies involving human research participants and Sex and Gender in Research.

Reporting on sex and gender	The discovery cohort included participants from the UK Biobank which consist of 502,411 adult participants aged between 40-69 years; with 54.4% females. The case-control discovery GWAS using UKB data for BCC included 307,684 participants (20,791 cases and 286,893 controls) and for SCC, 294,294 (7,402 SCC cases and 286,892 controls) individuals of European ancestry.
Population characteristics	Age, sex, and the first ten principal components were adjusted for in the GWAS.
Recruitment	This study did not directly recruit participants, but it used already collected data in other population based cohorts e.g. the UK Biobank for the two primary traits of interest; BCC and SCC, QSkin, Canadian Longitudinal Study on Aging, where participants were randomly recruited from the general population, and followup for disease outcomes. Details are included in the Methods and Supplementary Information.
Ethics oversight	This study was approved by the Human Research Ethics Committee (HREC) at QIMR Berghofer Medical Research Institute. The following cohorts were approved by the respective ethics committees; The UK Biobank (National North West Multi-Centre Research Ethics Committee), 23andMe Inc. (Association for the Accreditation of Human Research Protection Program), QSkin (HREC at QIMR Berghofer), and Canadian Longitudinal Study on Aging by Canadian Institutes of Health Research.

Note that full information on the approval of the study protocol must also be provided in the manuscript.

Field-specific reporting

Please select the one below that is the best fit for your research. If you are not sure, read the appropriate sections before making your selection.

☒ Life sciences ☐ Behavioural & social sciences ☐ Ecological, evolutionary & environmental sciences

For a reference copy of the document with all sections, see nature.com/documents/nr-reporting-summary-flat.pdf

Life sciences study design

All studies must disclose on these points even when the disclosure is negative.

Sample size	In the case-control GWAS using UKB data for BCC we included 307,684 participants (20,791 cases and 286,893 controls) and for SCC, 294,294 (7,402 SCC cases and 286,892 controls) individuals of European ancestry. Sample size size was chosen based on the number of participants of European ancestry with controls having no history of any cancer. With a sample size of > 200,000 this makes it a well powered GWAS.
Data exclusions	Individuals were excluded based on; non-European ancestry, high genotype missingness and relatedness (where applicable). Genetic variants were excluded based on; low call rate, minor allele frequency (<1%), and imputation quality score.

Replication	The discovery MTAG results (loci) were replicated in a large independent cohort of 23andMe Inc (USA). All the publicly available data used (e.g. melanoma, smoking, etc) had been previously replicated. The PRS was also validated before applying it in the target cohort.
Randomization	Randomisation was not applicable in this study because it was not a Randomised Control Trail (RCT). We used cohort data from observational studies. Randomisation is not necessary in GWAS analyses.
Blinding	Blinding is not necessary in GWAS analyses. This was not an RCT.

Reporting for specific materials, systems and methods

We require information from authors about some types of materials, experimental systems and methods used in many studies. Here, indicate whether each material, system or method listed is relevant to your study. If you are not sure if a list item applies to your research, read the appropriate section before selecting a response.

Materials & experimental systems

n/a	Involved in the study	
☒	☐	Antibodies
☒	☐	Eukaryotic cell lines
☒	☐	Palaeontology and archaeology
☒	☐	Animals and other organisms
☒	☐	Clinical data
☒	☐	Dual use research of concern

Methods

n/a	Involved in the study	
☒	☐	ChIP-seq
☒	☐	Flow cytometry
☒	☐	MRI-based neuroimaging
